# Cardiovascular risk among health professionals

**DOI:** 10.47626/1679-4435-2022-204

**Published:** 2023-02-13

**Authors:** Ricardo Moreira Martins

**Affiliations:** Vice President - Southern region, Associação Nacional de Medicina do Trabalho (ANAMT) 2019/2022-2023/2025; specialization in Cardiology from Instituto de Cardiologia - Fundação Universitária de Cardiologia (IC-FUC/RS); specialization in Occupational Medicine, ANAMT/ Associação Médica Brasileira (AMB); member of Câmara Técnica de Medicina do Trabalho of Conselho Regional de Medicina do Rio Grande do Sul (CREMERS); director of Professional Practice, Associação Médica do Rio Grande do Sul (AMRIGS)

The main cause of morbidity and mortality in the world is cardiovascular disease. In 2015, it was responsible for 17.7 million deaths (31% of deaths worldwide), of which 7.4 million were due to coronary disease and 6.7 million to stroke.^1^ The risk factors for cardiovascular disease can be classified as modifiable (dyslipidemia, smoking, alcoholism, diabetes mellitus, systemic arterial hypertension, obesity, excessive daytime sleepiness, depression, stress, obstructive sleep apnea, and sedentary lifestyle) or non-modifiable (age, sex, heredity, and race).

In the work environment, health professionals are exposed daily to stress, deteriorating public structures, poor service management, work overload, double shifts, social pressure, underemployment, low wages, and hopelessness, all of which contribute to professional frustration and, ultimately, to occupational disease. Health units are environments of intense work and continuous exposure to suffering, pain, anguish, and death, which often leads to neglect of self-care regarding physical activity, diet, smoking cessation, alcohol use, and coping strategies.

Occupational medicine is primarily preventive, and it is the occupational physician’s duty to proactively search for individual and collective data from workers, prioritize epidemiological monitoring, and implement preventive measures. Several Brazilian publications have evaluated cardiovascular risk factors among health team members, mainly regarding the prevalence of arterial hypertension, diabetes mellitus, dyslipidemia, sedentary lifestyle, obesity, diet, and smoking, finding that they are significant in this population.

For the most part, these studies confirm that there is a significant prevalence of cardiovascular risk factors among health professionals. Many of these factors are associated with the patients’ sociodemographic characteristics and lifestyle. Lifestyle-related factors, if identified, can usually be minimized through educational programs provided by Specialized Services in Safety Engineering and Occupational Medicine (SESMT).

One primary prevention measure is behavioral modification, which includes setting goals, data on individual and collective health conditions, clear guidance about health impacts, action planning, self-monitoring of health behaviors in individual consultations, group consultations, and printed materials. In addition to education, prevention measures also include developing policies to promote physical activity and reduce obesity, eg, healthy environments that favor walking and cycling, natural areas at the workplace for physical activity, and guidance about caloric restriction.

The increasing prevalence of professional burnout, which occurs mainly among women, is characterized by pain, headache, neck pain, and low back pain, can limit normal activities and affect aspects such as vitality. Since these complaints occur in situations involving repetitive work, non-ergonomic positions, and high-speed movements, such as in trauma care sectors, it can be concluded that health professionals are vulnerable. The agendas of SESMT and health care institutions should move towards collective awareness and the creation of a warning and protection network against worsening depressive syndromes and suicide.

Underreporting is a reality: given that these professionals are knowledgeable about health problems and their treatments, they frequently omit information about them to the medical service, using self-medication as a coping strategy. The difficulties SESMT professionals face in identifying health problems are due to a lack of sentinel events, which are fundamental for individual and collective action.

According to estimates, 23 million people will die from cardiovascular disease in 2030, thus it will remain the leading cause of death worldwide.^2^ The World Health Organization estimates that three-quarters of cardiovascular deaths can be prevented through lifestyle change.

The most important behavioral factors associated with heart disease and stroke are poor diet, sedentarism, and tobacco and alcohol use, which together account for 80% of coronary artery and cerebrovascular disease cases. As an example, in the study “Comparison of Cardiovascular Risk Factors in Different Areas of Health Care Over a 20-Year Period”^2^ it is described:

“In general, there was an increase in the prevalence of CVRFs [cardiovascular risk factors] assessed in the population studied, despite the individuals’ technical knowledge of these risk factors. There was an increase in excessive weight gain, SAH [systemic arterial hypertension], and dyslipidemia among physicians and dentists. Excessive weight gain, increased prevalence of SAH, and decreased sedentarism were observed among pharmacists. Excessive weight gain and alcohol consumption was observed among nurses. Nutritionists showed an increased prevalence of dyslipidemia.”

“The analysis of these compiled data, considering only the number of CVRFs that had a positive or negative variation over the 20 years, suggests more CVRF progression among physicians and dentists and less CVRF progression among nutritionists.”^2^

Therefore, primary cardiovascular prevention strategies are indispensable for promoting worker health.


**Ricardo Moreira Martins** Vice President - Southern region,
Associação Nacional de Medicina do Trabalho (ANAMT)
2019/2022-2023/2025; specialization in Cardiology from Instituto de Cardiologia -
Fundação Universitária de Cardiologia (IC-FUC/RS);
specialization in Occupational Medicine, ANAMT/ Associação
Médica Brasileira (AMB); member of Câmara Técnica de Medicina
do Trabalho of Conselho Regional de Medicina do Rio Grande do Sul (CREMERS);
director of Professional Practice, Associação Médica do Rio
Grande do Sul (AMRIGS).

